# Validating administrative data to identify complex surgical site infections following cardiac implantable electronic device implantation: a comparison of traditional methods and machine learning

**DOI:** 10.1186/s13756-022-01174-z

**Published:** 2022-11-10

**Authors:** Elissa Rennert-May, Jenine Leal, Matthew K. MacDonald, Kristine Cannon, Stephanie Smith, Derek Exner, Oscar E. Larios, Kathryn Bush, Derek Chew

**Affiliations:** 1 Department of Medicine, University of Calgary, Calgary, AB Canada; 2 Department of Community Health Sciences, University of Calgary, Calgary, AB Canada; 3 O’Brien Institute for Public Health, University of Calgary, Calgary, AB Canada; 4 Department of Microbiology, Immunology and Infectious Diseases, University of Calgary, Calgary, AB Canada; 5 Snyder Institute for Chronic Diseases, University of Calgary, Calgary, AB Canada; 6 Infection Prevention and Control, Alberta Health Services, Calgary, AB Canada; 7 Libin Cardiovascular Institute, University of Calgary, Calgary, AB Canada; 8 Department of Medicine, University of Alberta, Edmonton, AB Canada; 9 Department of Cardiac Sciences, University of Calgary, Calgary, AB Canada; 10 Department of Pathology and Laboratory Medicine, University of Calgary, Calgary, AB Canada

**Keywords:** Surveillance, Surgical site infections, Administrative data

## Abstract

**Background:**

Cardiac implantable electronic device (CIED) surgical site infections (SSIs) have been outpacing the increases in implantation of these devices. While traditional surveillance of these SSIs by infection prevention and control would likely be the most accurate, this is not practical in many centers where resources are constrained. Therefore, we explored the validity of administrative data at identifying these SSIs.

**Methods:**

We used a cohort of all patients with CIED implantation in Calgary, Alberta where traditional surveillance was done for infections from Jan 1, 2013 to December 31, 2019. We used this infection subgroup as our “gold standard” and then utilized various combinations of administrative data to determine which best optimized the sensitivity and specificity at identifying infection. We evaluated six approaches to identifying CIED infection using administrative data, which included four algorithms using International Classification of Diseases codes and/or Canadian Classification of Health Intervention codes, and two machine learning models. A secondary objective of our study was to assess if machine learning techniques with training of logistic regression models would outperform our pre-selected codes.

**Results:**

We determined that all of the pre-selected algorithms performed well at identifying CIED infections but the machine learning model was able to produce the optimal method of identification with an area under the receiver operating characteristic curve (AUC) of 96.8%. The best performing pre-selected algorithm yielded an AUC of 94.6%.

**Conclusions:**

Our findings suggest that administrative data can be used to effectively identify CIED infections. While machine learning performed the most optimally, in centers with limited analytic capabilities a simpler algorithm of pre-selected codes also has excellent yield. This can be valuable for centers without traditional surveillance to follow trends in SSIs over time and identify when rates of infection are increasing. This can lead to enhanced interventions for prevention of SSIs.

**Supplementary Information:**

The online version contains supplementary material available at 10.1186/s13756-022-01174-z.

## Background

Surgical site infections (SSI) following cardiac implantable electronic device (CIED) implantation are hospital acquired infections (HAIs) that pose a substantial burden on our healthcare system [1, 2]. The annual rate of CIED implantations has increased over the past several decades due to the increasing need for pacemaker therapy in an aging population, and expanding indications for cardiac resynchronisation therapy (CRT) and prophylactic implantable cardioverter defibrillators (ICDs). Notably, the rate of CIED infections is outpacing the increase in implantation rate [3].

CIED infections can include local infections such as pocket infections, but can also include more severe systemic infections including bloodstream infections and infective endocarditis [4]. Randomized controlled trial data suggest relatively low baseline rates of CIED infection (i.e. approximately 1%) [5]. However, studies using large population-based observational data that better reflect everyday practice demonstrate CIED infection rates as high as 4% [6]. The consequence of CIED infection is increased patient morbidity and mortality. Additionally, CIED infections are associated with substantial medical resource use and costs due to repeat hospitalizations, multiple surgeries for device removal and possible re-implantation, and lengthy hospital stays for intravenous antibiotics [7, 8].

Given the impact of these infections, it is essential to perform surveillance of CIED infections within healthcare systems. Surveillance has been identified as a core component for any Infection Prevention and Control (IPC) program by international health organizations, which may include post-operative patient surveillance in the outpatient, emergency departments or inpatient setting, with detailed patient chart review, and tracking of microbiology results [9]. Unfortunately, comprehensive IPC surveillance programs for CIED infections can be costly and time-consuming, and require extensive human resources. Particularly in smaller centers without extensive funding for IPC programs this type of robust surveillance may not be practical.

An alternative method to identify CIED infections is the use of administrative data, which may be less expensive and resource intensive. However, the validity of such data for infection surveillance has been questioned and may be dependent on the type of HAI for identification [10]. A systematic review of administrative data for SSI surveillance found that across 34 studies, sensitivity and positive predictive values (PPV) only performed moderately, which may be due to pooling heterogeneous study populations undergoing very different surgical operations [11]. CIED infections were not one of the SSI included in that review.

Given the absence of studies assessing the use of administrative data for CIED infection surveillance, our study sought to validate administrative codes to robustly identify CIED infections compared to a gold standard of comprehensive IPC surveillance with chart review. A secondary objective was to determine the best administrative data approach for CIED infection identification using conventional selection of an unweighted set of preselected codes, or machine learning methods [12].

## Methods

The study protocol was developed based on the modified Standards for Reporting of Diagnostic Accuracy (STARD) criteria, and recommendations on the design and reporting of administrative data validation studies [13, 14].

### Study design and cohort

We identified a cohort of adults patients (i.e., age ≥ 18 years) in Calgary, Alberta who underwent de novo CIED implantation (including pacemaker (PM), ICD, or CRT) or generator replacement between January 1, 2013 and December 31, 2019. Among these patients undergoing CIED surgery, those developing a CIED infection within one year of surgery were identified through manual chart review enabled through a formal IPC Surveillance program. CIED infections were adjudicated using the Centers for Disease Control and Prevention/National Healthcare Safety Network (NHSN) standardized definitions for complex SSIs (i.e., deep or organ space) [15]. Superficial infections without involvement of the device or leads were not included as these types of infections are not an indication for CIED system removal and lead extraction [16]. This study focused on complex SSIs following CIED implantation given the substantial burden to health system (I.e., hospitalization, prolonged antibiotics, re-operation for system removal and reimplantation).These “gold standard” CIED infections were then used to validate infections identified through administrative coding data from the International Classification of Diseases-10^th^ revision in Canada (ICD-10-CA) and Canadian Classification of Health Intervention (CCI) administrative codes (ref https://www.cihi.ca/en/cci-coding-structure).

### Data sources

#### Paceart™

CIED implantations were identified using the PaceArt™ database, which is a repository of all device-related clinical encounters for any patient followed within the province of Alberta, Canada. As comprehensive IPC surveillance for CIED infections is not readily available across the province, we limited the base cohort to patients with CIED procedures performed within the Calgary zone. Calgary is an urban city with a population of approximately 1.5 million. Paceart™ contains information regarding indications for device implantation, type of device, date of operation and basic demographic information. Repeat procedures were censored in the two-year period from index surgical date to avoid double counting patient encounters that may be attributed to a single CIED infection (e.g. for a patient who underwent de novo pacemaker implantation requiring a lead revision 2 months later, only the initial implant was counted as an index procedural date).

#### Infection prevention and control

Complex SSI cases were obtained via collaboration with Alberta Health Services (AHS) Calgary Zone Infection Prevention & Control (IPC) (one IPC department completes surveillance for Calgary zone). Superficial infections were not collected. All patients with CIED implantation are actively followed for one year to determine if they develop infection using various sources including hospital admissions, chart review, and microbiology data. This surveillance is performed by a trained Infection Control Professional with AHS who has access to patient charts. Case identification follows NHSN definitions for eligibility and complex infection criteria. Infections that are identified are brought forth to a committee for case adjudication including the Infection Control Professional, Cardiac Electrophysiologists and Infectious Diseases physicians. Surveillance results are fed back to clinicians, and quality improvement strategies are implemented if CIED infection rates increase beyond acceptable levels. The importance of surveillance is recognized as an intervention which can lead to decreases in post-surgical infections. This IPC data set formed our reference (gold standard) CIED infection target variable, which was used to validate the administrative codes.

### AHS analytics

AHS is a single health system that services the entire province of Alberta, Canada. We utilized AHS analytics data which provides healthcare information on all Alberta residents with an Alberta Health Care Insurance Plan (> 99% of provincial coverage) in order to obtain information from the Discharge Abstract Database (DAD) for our patients in Calgary. This data was utilized to provide information on all hospital admissions and their associated ICD-10-CA and CCI codes (Additional file 1) for our baseline cohort, for one year after their initial CIED implantation or generator replacement. We assumed that all patients with a complex CIED infection would present to the Emergency Department or be admitted to hospital for management and that essentially all patients would require removal of their device.

### Statistical analyses

We identified the CCI codes published by Canadian Institute for Health Information (CIHI), which could potentially be used to identify CIED procedures (Additional file 1). These administrative codes were used to identify potential CIED infection cases through AHS analytics data. All patients in the base cohort were followed for one year from their initial CIED implantation or generator replacement to determine if they had the “infection” codes in subsequent hospital admissions.

We used three different pre-defined algorithms to track infection. First, we searched a combination of ICD-10-CA codes for infection including: infection following a procedure (T814), infection of an implantable cardiovascular or other device (T827, T857) or surgical procedure as the cause of an abnormal reaction (Y83), with the CCI codes listed in Additional file 1 which represent CIED procedures (Algorithm 1). Second, we assessed an approach from a recent publication using non-validated ICD-10-CA codes to track infection: Infection of an implantable cardiovascular or other device (T827, T857), infective endocarditis (I330, I339, I38, I398), or cellulitis of the chest wall or other unspecified site (L0330, L0339, L038, L039) (Algorithm 2) [8]. Third, we used an approach of only CCI codes related to implantation/removal of CIEDs as follows 1.YY.54.^, 1.YY.54.LA-NJ, 1.HZ.55.^, 1.HD.53.^, 1.HD.53.GR-JA, 1.HD.54.^, 1.HD.54.GR-JA, 1.HD.55.^, 1.HD.55.GP-JB, or 1.HD.55.GR-JA (Algorithm 3) [17]. For all three approaches above, we used standard epidemiological methods to calculate sensitivity, specificity, PPV, negative predictive value (NPV) and area under the receiver operating characteristic (ROC) curve (AUC) metrics for the administrative code classification predictions.

An additional analysis was conducted where we trained a machine learning model to determine the most appropriate codes to identify infection rather than the traditional pre-identification of codes as outlined above. Logistic regression models were trained using Python and the scikit-learn library [17]. Due to the high class imbalance of the dataset (133:1 occurrence of infection) the class weights were scaled to be inversely proportional to frequency when training the model. L1 regularization was used on the model coefficients during training for its feature selection capability and to avoid overfitting the model [18]. Due to the limited number of infection examples a stratified cross validation strategy was applied to evaluate the models. A test set was not used because the primary aim was a comparison of the different approaches. The outer cross validation loop consisted of five random stratified folds of the full dataset. The same strategy was used to evaluate the performance of the traditional approaches. For the logistic regression models a nested inner training loop consisted of a grid search for regularization strength using three stratified folds of the training data split.

Ultimately, we evaluated two logistic regression models: a model trained on ICD-10-CA and CCI codes with regularization C = 0.6 (*ICD & CCI Model*), as well as a model trained on only ICD-10-CA codes with a regularization C = 0.4 (*ICD Model*). Finally, we simply looked for the ICD-10-CA code T827 given its definition of “infection of an implantable cardiovascular device”.

Following validation of administrative codes, we compared temporal trends between the CIED infections identified via gold standard IPC surveillance and those identified using the optimal administrative approach. Analysis was completed using Python version 3.9.12 and Scikit-learn version 1.1.1. Ethics for this study was obtained from the University of Calgary Health Research Ethics Board (REB20-2186).

## Results

### Study cohort

Between January 1 2013 and December 31 2019, there were 3536 CIED procedures performed, and a total of 5631 hospitalizations. Among these 3536 procedures, there were 42 infections (1.2%) identified through comprehensive IPC surveillance.

Baseline characteristics of the overall cohort and those with SSI’s can be found in Table 1. The most common procedure was PM insertion (72%). However, SSIs following PM insertion only accounted for 45% overall. ICDs which accounted for 20% of all device implantations accounted for 36% of SSIs.

**Table 1:** Baseline characteristics of the Paceart cohort and infection subgroup

	Total Paceart Cohort	Infection Subgroup
Number of Patients & Device Implants (Total N)	3,536	42
Age In Years, Mean (SD)	73.6 (14.6)	67.0 (16.0)
Sex		
Male	2,253 (64%)	29 (69%)
Device Type		
PM N (% Total N)	2,551 (72%)	19 (45%)
ICD N (% Total N)	722 (20%)	15 (36%)
CRT N (% Total N)	263 (7%)	8 (19%)
Generator Replacement N (% Total N)	676 (19%)	4 (10%)

PM, pacemaker; ICD, intracardiac device; CRT, cardiac resynchronization therapy

### Administrative data algorithms for CIED infection identification

**Fig. 1:**
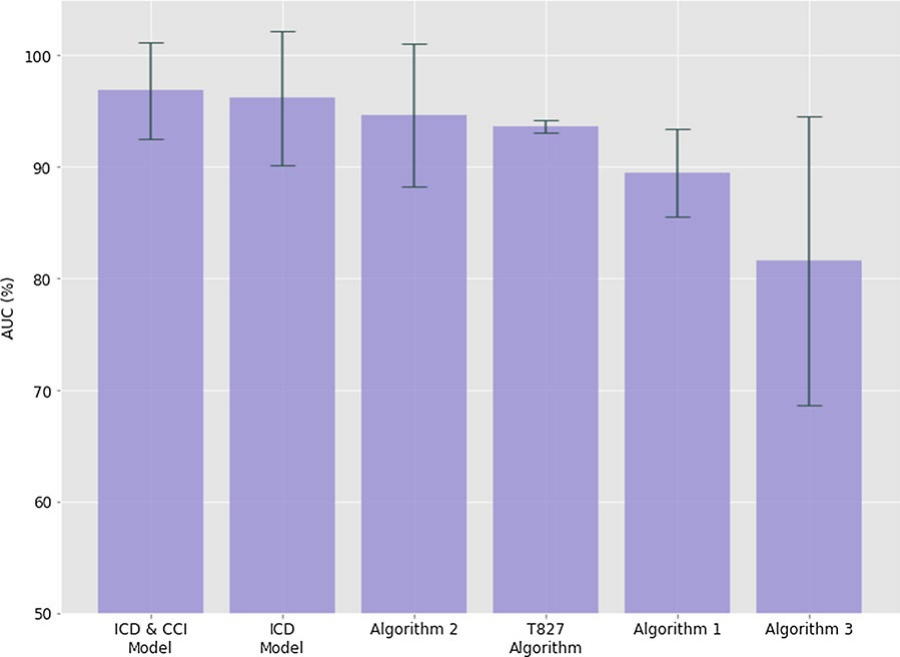
Plot of AUC metric for SSI classification with bars representing 95% confidence

**Fig. 2:**
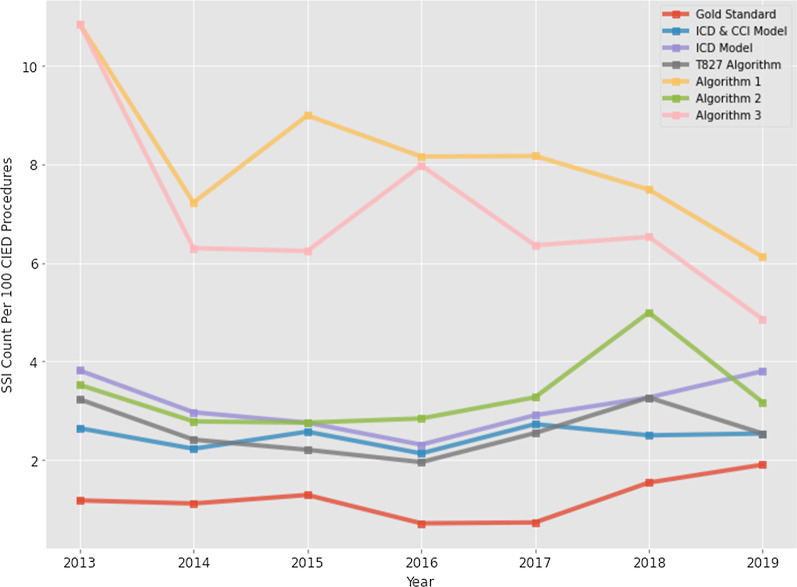
Plot of yearly predicted and confirmed SSI per 100 CIED procedures in Calgary

All of the algorithms to identify infections utilizing administrative codes performed well. Sensitivity, specificity, PPV, NPV and AUC for each approach can be found in Table 2. Ranked based on AUC, the highest performing approach was the ICD/CCI code based logistic regression model with an average AUC of 96.8%. The ICD code only model followed closely behind with an average AUC of 96.1%. A visual representation of each AUC can be found in Fig. 1. Trends in SSIs over time identified by administrative data using all included approaches as well as the “gold standard” cohort of SSIs identified by IPC can be seen in Fig. 2. All approaches overpredict the number of SSIs, as seen in the PPV metric. The improved proximity of the trendline profile and values to the gold standard is evident for the top performing models/algorithms. While sensitivity and specificity are excellent, the PPV is low. This is due to the fact that PPV is affected by the prevalence of the “disease,” in this case the CIED infection. When prevalence is low the PPV will also be low irrespective of the sensitivity and specificity. However, the sensitivity and specificity reflect the ability to discriminate between a true positive and a true negative result. These characteristics are what is required to ensure a test or algorithm is useful for diagnosis [19].

**Table 2:** Sensitivity, specificity, positive predictive value and negative predictive value for each approach

	Area Under Curve (ROC)	Sensitivity	Specificity	Negative predictive value	Positive predictive value
Algorithms (Traditional pre-selected codes)					
Algorithm 1	0.894 ± 0.04	0.833 ± 0.08	0.955 ± 0.005	0.999 ± 0.001	0.124 ± 0.016
Algorithm 2	0.946 ± 0.064	0.906 ± 0.128	0.986 ± 0.001	0.999 ± 0.001	0.325 ± 0.049
Algorithm 3	0.816 ± 0.13	0.669 ± 0.261	0.962 ± 0.004	0.997 ± 0.002	0.115 ± 0.04
Machine learning models					
ICD Model	0.961 ± 0.06	0.903 ± 0.129	0.987 ± 0.009	0.999 ± 0.001	0.378 ± 0.133
ICD & CCI Model	0.968 ± 0.043	.9 ± 0.13	0.99 ± 0.004	0.999 ± 0.001	0.416 ± 0.047
Unique Code					
T827	0.936 ± 0.006	0.881 ± 0.009	0.991 ± 0.005	0.999 ± 0.0	0.428 ± 0.119

Average metrics ± 95% confidence interval based on fivefold cross validation

**Fig. 3:**
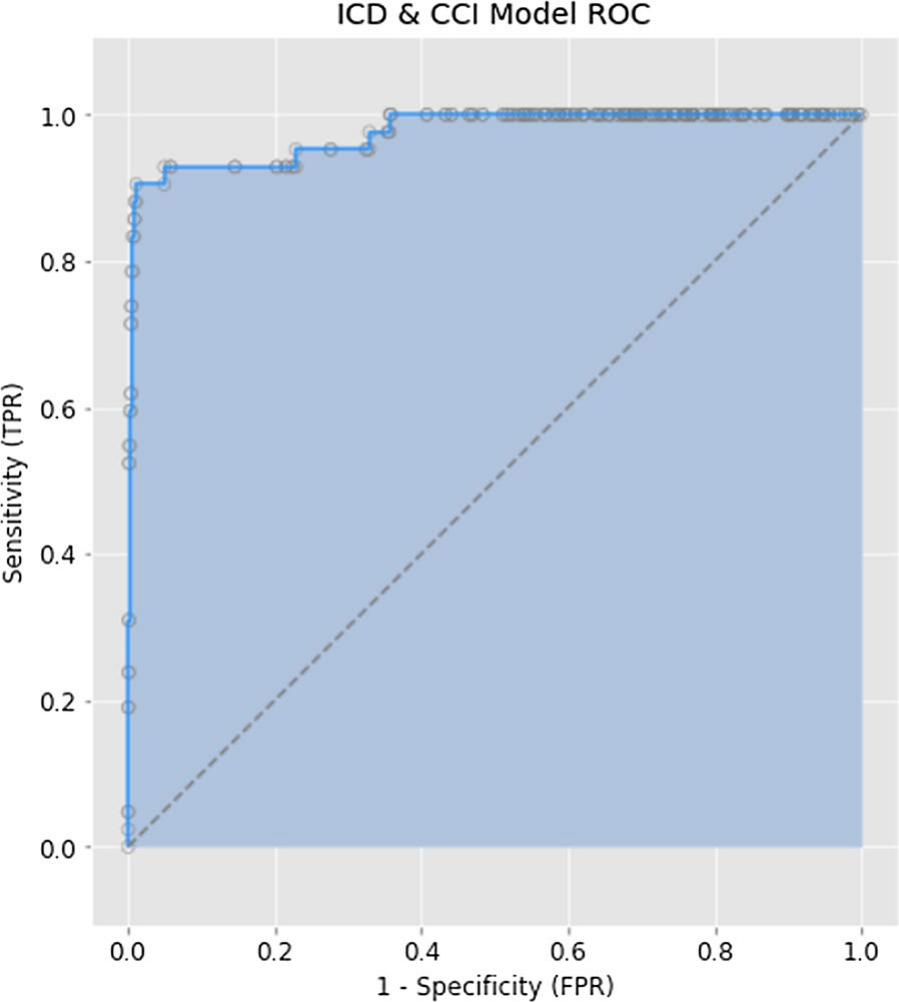
ROC curve for highest performing machine learning model (*ICD & CCI Model*)

The best performing machine learning model was comprised of 48 non-zero ICD and CCI codes, which are shown along with their weights in Table 3. Training with a high regularization strength of 0.1 reduces this list to the top 5 most predictive codes in order of importance: T827 (Infection and inflammatory reaction due to other cardiac and vascular device, implants and grafts), Y831 (Surgical operation with implant of artificial internal device as the cause of abnormal reaction of the patient, or of later complication, without mention of misadventure at the time of the procedure), 1IS53 and 1IS53GR (Implantation of internal device, vena cava (superior and inferior), percutaneous transluminal approach), 1HZ53 (Implantation of an internal device, heart). The ROC curve for this model can be visualized in Fig. 3. All trained logistic regression models prioritized T827 as the most important indicator of SSI regardless of regularization.

**Table 3:** Non-zero coefficients of ICD and CCI codes in best performing model

ICD or CCI Code	Coefficient	Description
T827	5.796482	Infection and inflammatory reaction due to other cardiac and vascular devices, implants and grafts
Z4501	4.644129	Encounter for adjustment and management of cardiac pacemaker
K635	3.679172	Polyp of colon
A41	− 3.49229	Other sepsis
Y831	2.765935	Surgical operation with implant of artificial internal device as the cause of abnormal reaction of the patient, or of later complication, without mention of misadventure at the time of the procedure
I50	− 2.74143	Heart failure
1HZ53GRFU	2.560292	Implantation of internal device, heart NEC, cardiac resynchronization therapy defibrillator, percutaneous transluminal approach
Z9500	− 2.49368	Presence of cardiac pacemaker
1HZ55	2.484518	Removal of device, heart NEC
I25	2.328524	Chronic ischemic heart disease
Z950	2.304491	Presence of cardiac pacemaker
2NK70BA	1.828463	Inspection, small intestine, using endoscopic per orifice (or via stoma)
T81	1.564795	Complications of procedures, not elsewhere classified
1HD55	1.499127	Removal of device, endocardium
N189	1.459065	Chronic kidney disease, unspecified
T8279	1.440367	Infection and inflammatory reaction due to other and unspecified cardiac and vascular devices, implants and grafts
1IS53	1.412757	Implantation of internal device, vena cava (superior and inferior)
1YY87	− 1.34526	Excision partial, skin of surgically constructed sites
Y832	− 1.21964	Surgical operation with anastomosis, bypass or graft as the cause of abnormal reaction of the patient, or of later complication, without mention of misadventure at the time of the procedure
I44	− 1.19129	Atrioventricular and left bundle-branch block
T810	1.143426	Haemorrhage and haematoma complicating a procedure, not elsewhere classified
L022	1.066629	Cutaneous abscess, furuncle and carbuncle of trunk
1IS53GRLF	0.981519	Implantation of internal device, vena cava (superior and inferior), non-tunnelled central venous catheter, percutaneous transluminal approach
1HZ53	0.978848	Implantation of internal device, heart NEC
E119	0.851433	Type 2 diabetes mellitus without complications
T821	− 0.81777	Mechanical complication of cardiac electronic device
1HB55LAJA	0.723747	Removal of device, epicardium, of pacemaker/defibrillator leads and open approach
N39	− 0.5671	Other disorders of urinary system
T813	0.562346	Disruption of wound, not elsewhere classified
I4800	− 0.55427	Paroxysmal atrial fibrillation
1HZ55GPFS	0.449912	Removal of device, heart NEC, cardioverter/defibrillator, percutaneous transluminal approach
2NK70BABJ	0.435566	Inspection, small intestine, using endoscopic per orifice (or via stoma) and colonoscope
1GV52	− 0.35724	Drainage, pleura
1IS53GR	0.328088	Implantation of internal device, vena cava (superior and inferior), percutaneous transluminal approach
R57	− 0.22109	Shock, not elsewhere classified
N17	− 0.21512	Acute kidney failure
1HB55	0.162474	Removal of device, epicardium
1HZ53GRNM	0.149551	Implantation of internal device, heart NEC, single chamber rate responsive pacemaker, percutaneous transluminal approach
B956	0.134421	Staphylococcus aureus as the cause of diseases classified elsewhere
Z515	− 0.12278	Encounter for palliative care
I42	0.110606	Cardiomyopathy
T82	− 0.09459	Complications of cardiac and vascular prosthetic devices, implants and grafts
1HB55LA	0.087532	Removal of device, epicardium, of pacemaker/defibrillator leads
1NM87BA	0.07593	Excision partial, large intestine, endoscopic per orifice approach
1YY87LA	− 0.05065	Excision partial, skin of surgically constructed sites, using open (excisional) approach
I48	− 0.04421	Atrial fibrillation and flutter
1GV52HA	− 0.00726	Drainage, pleura, using percutaneous (needle) approach

## Discussion

We found that between January 1, 2013 and December 31, 2019 there were a total of 3,536 CIED implantations including generator replacements in Calgary zone. Of these, 42 (1.2%) developed a complex SSI. While PM insertions were the most common CIED procedure, ICD and CRT implantation accounted for a disproportionately greater amount of SSIs compared to their implantation rate, which is consistent with previous literature [8]. We found that administrative data was able to perform very well (AUC > 90%) at identifying SSIs post CIED implantation, including both pre-selected ICD/CCI code algorithms and a machine learning approach. Trends in SSIs were similar between the traditionally collected data, and the best performing administrative data approaches.

Our findings are consistent with previous recent work done on validating administrative data for SSIs. Prior work done on SSIs following hip and knee replacement found that pre-selected ICD code algorithms using administrative data were able to achieve sensitivity > 80% and specificity close to 100% compared to a gold standard of SSI collection [20, 21]. Previous studies exploring HAIs in general have examined the use of administrative data to identify these infections. The results have been inconsistent with some studies reporting that administrative data using ICD codes was of limited benefit in identifying HAIs [22]. The previously mentioned systematic review [11] which encompassed 57 studies, including 34 looking specifically at SSIs, found a wide range of sensitivity for using ICD codes to identify HAI ranging from 10 to 100% and PPV from 11 to 95% suggesting that administrative data was extremely variable at identifying infections [11]. However, it is important to note that many of those studies were done using ICD-9 revision codes as opposed to the more granular ICD-10 codes [23] which likely improve the yield of administrative data at identifying infections. Our current findings in corroboration with the aforementioned recent studies on SSIs do suggest that administrative data can be an effective tool at identifying SSIs.

There is very little literature available validating the use of administrative data for identification of SSIs following CIED implantation and comparing the use of pre-selected ICD/CCI codes and machine learning. A recent study by Mull et al. did use a unique method of combining structured and text data in Veterans Health Facilities electronic medical records in order to identify SSIs following CIED implantation [24]. In their validation logistic regression model they were able to achieve an AUC of 90%. However, this type of work would rely on the availability of data via an electronic medical record system which is not always available at centers. Furthermore, the results have limited generalizability as collected data fields may vary depending on the electronic medical system deployed in a particular healthcare system.

Machine learning is becoming increasingly recognized as an important tool for infection identification and control. A review of 52 studies looked at the ability of machine learning technique to detect or predict sepsis, HAIs (including SSIs) and other infections. However, they found that understanding and interpretability of the models was rarely addressed [25]. Our study provides a clear application and interpretation of a machine learning approach for identifying SSIs following CIED implantation contributing to the literature. Our study also demonstrated that one unique code T827 still had a very high AUC (> 90%), and therefore in situations where it may not be practical to compute a weighted total of 48 codes this could be a simpler alternative. Nevertheless, validation of administrative codes to identify CIED infection lays the foundation to adapt these algorithms in clinical practice. Potential applications include fully automated or semi-automated surveillance supplemented by traditional IPC methods [26]; however, implementation will depend on local health system needs, acceptability and resources.

Our study has several additional strengths. First, our results are generalizable as the current study validates the use of widely available administrative data—ICD-10 and CCI codes, for identification of SSIs following CIED implantation. In settings that do not use CCI codes for procedural reporting, our study provides several algorithms based on ICD-10 codes alone. Second, our work compares different methods of more traditional pre-selected data codes and machine learning approaches to logistic regression in order to identify the most optimal way to use administrative data for infection detection. Several algorithms had comparable test characteristics, which provides options for implementation depending on the needs and resources of regional health systems. For example, the algorithms using only ICD-10 codes would be applicable outside Canada, where CCI codes are not available. Finally, our study is facilitated by the availability of comprehensive IPC surveillance for CIED infections s which provides a “gold standard” to use for validation of the administrative data.

The limitations of this work must also be considered. The number of identified infections was very small given that the availability of the gold standard infection data was restricted to one city. However, this population-based study included a cohort of all CIED implantations from a large regional health zone comprised of urban, community and rural hospitals, which improves the external validity of our study. Another limitation was that IPC surveillance identified infections within one year post procedure. While most infections would occur during this timeframe any infections that occurred very late (beyond one year) would have been missed.

## Conclusions

While traditional IPC surveillance is the current gold standard for identifying CIED infections, this comprehensive approach requires substantial time and resources, which may not be feasible, especially in smaller hospital centers. The current study validates a method using administrative data for accurate identification of complex SSIs following CIED implantation. While the best performance came from the machine learning models, pre-selected ICD codes also performed very well (AUCs > 90%). At centers with limited data analytic capacity where machine learning applications and validation would not be feasible, the more traditional approach of pre-selected ICD-10 codes could be used to track SSIs and trends over time. Our findings set the foundation for more accessible surveillance of CIED infections and identification of outlier infection rates compared to historic trends, which can lead to targeted interventions for prevention of SSIs.

## Supplementary Information

**Additional file 1**. CCI Codes representing CIED implantation.

## Data Availability

The data that support the findings of this study are available from Alberta Health Services but restrictions apply to the availability of these data, which were used under license for the current study, and so are not publicly available. Data are however available from the authors upon reasonable request and with permission of Alberta Health Services and appropriate ethics.

## Abbreviations

CIED: Cardiac implantable electronic devices
PM: Pacemaker
ICD: Implantable cardioverter defibrillator
CRT: Cardiac resynchronization therapy
IPC: Infection prevention and control
SSI: Surgical site infection
HAI: Hospital acquired infection
AHS: Alberta Health Services
DAD: Discharge Abstract Database
ICD: International Classification of Diseases
CCI: Canadian Classification of Health Interventions
AUC: Area under the receiver operating characteristic Curve
ROC: Receiver operating characteristic
PPV: Positive predictive value
NPV: Negative predictive value
NHSH: National Healthcare Safety Network
CIHI: Canadian Institute for Health Information
