# Supplementary material for: Validating administrative data to identify complex surgical site infections following cardiac implantable electronic device implantation: a comparison of traditional methods and machine learning
Source: Antimicrob Resist Infect Control. 2022 Nov 10;11:138. doi: 10.1186/s13756-022-01174-z (PMC9650806; doi:10.1186/s13756-022-01174-z)
Supplement: Supplementary file 1 — Additional file 1. CCI Codes representing CIED implantation. [file 13756_2022_1174_MOESM1_ESM.docx]

**Additional File 1**

| **CCI** | **Procedure description** |
| --- | --- |
| **CIHI PACE procedures** | |
| 1.HB.53.^^ | Implantation of epicardium |
| 1.HB.54.^^ | Management of internal device, epicardium |
| 1.HB.55.^^ | Removal of epicardium |
| 1.HD.53.^^ | Implantation endocardium |
| 1.HD.54.^^ | Management of endocardium |
| 1.HD.55.^^ | Removal of endocardium |
| 1.HZ.53.GR-FR | Implantation of CRT pacemaker |
| 1.HZ.53.GR-FS | Implantation of cardioverter-defibrillator |
| 1.HZ.53.GR-FU | Implantation of CRT defibrillator |
| 1.HZ.53.GR-NK | Implantation of dual chamber rate responsive pm |
| 1.HZ.53.GR-NL | Implantation of fixed rate pm |
| 1.HZ.53.GR-NM | Implantation of single chamber rate responsive PM |
| 1.HZ.53.HA-FS | Percutaneous approach to cardioverter/defibrillator |
| 1.HZ.53.LA-FR | Open approach to CRT pm |
| 1.HZ.53.LA-FS | Open approach to cardioverter/defibrillator |
| 1.HZ.53.LA-FU | Open approach to CRT defibrillator |
| 1.HZ.53.LA-NK | Open approach to dual chamber rate responsive PM |
| 1.HZ.53.LA-NL | Open approach to fixed rate PM |
| 1.HZ.53.LA-NM | Open approach to single chamber rate responsive pm |
| 1.HZ.53.QA-NK | Open subxiphoid approach to dual chamber rate responsive pm |
| 1.HZ.53.QA-NL | Open subxiphoid approach to fixed rate pm |
| 1.HZ.53.QA-NM | Open subxiphoid approach to single chamber rate responsive Pm |
| 1.HZ.53.SY-FR | Combined open and percutaneous approach to CRT pm |
| 1.HZ.53.SY-FS | Combined open and percutaneous approach to cardioverter/defibrillator |
| 1.HZ.53.SY-FU | Combined open and percutaneous approach to CRT defibrillator |
| 1.HZ.55.GP-^^ | Removal of device, heart NEC, percutaneous transluminal approach |
| 1.HZ.55.LA-FR | Removal of device, heart NEC, cardiac resynchronization therapy pacemaker [CRT, CRT-P, Biventricular pacemaker], open approach (e.g. sternotomy) |
| 1.HZ.55.LA-FS | Removal of device, heart NEC, cardioverter/defibrillator [AICD], open approach (e.g. sternotomy) |
| 1.HZ.55.LA-FU | Removal of device, heart NEC, cardiac resynchronization therapy defibrillator [CRT-D, BiV-ICD], open approach (e.g. sternotomy) |
| 1.HZ.55.LA-NK | Removal of device, heart NEC, dual chamber rate responsive pacemaker, open approach (e.g. sternotomy) |
| 1.HZ.55.LA-NL | Removal of device, heart NEC, fixed rate pacemaker, open approach (e.g. sternotomy) |
| 1.HZ.55.LA-NM | Removal of device, heart NEC, single chamber rate responsive pacemaker, open approach (e.g. sternotomy) |
| 1.HZ.55.QA-^^ | Removal of device, heart NEC, open subxiphoid approach |
| 1.YY.54.LA-FS | Management of internal device, skin of surgically constructed sites, of cardioverter or defibrillation device using open (subcutaneous) approach |
| 1.YY.54.LA-NM | Management of internal device, skin of surgically constructed sites, of cardiac pacemaker battery/generator using open (subcutaneous) approach 1 |
| 1.YY.55.LA-FS | Removal of internal device, skin of surgically constructed sites, of cardioverter or defibrillation device using open (subcutaneous) approach 1 |
| 1.YY.55.LA-NM | Removal of internal device, skin of surgically constructed sites, of cardiac pacemaker battery/generator using open (subcutaneous) approach |

*Canadian Institute for Health Information. Canadian Classification of Health Interventions (CCI). 2015.

*Methodology Notes for Hospitalized Surgical Site Infections. CIHI. 2017
